# Retraction: Machine Learning-Based Prediction of COVID-19 Prognosis Using Clinical and Hematologic Data

**DOI:** 10.7759/cureus.r213

**Published:** 2026-01-02

**Authors:** Fatemah O Kamel, Rania Magadmi, Sulafah Qutub, Maha Badawi, Mazen Badawi, Tariq A Madani, Areej Alhothali, Ehab A Abozinadah, Duaa M Bakhshwin, Maha H Jamal, Abdulhadi S Burzangi, Mohammed Bazuhair, Hussamaldin Alqutub, Abdulaziz Alqutub, Sameera M Felemban, Fatin Al-Sayes, Soheir Adam

**Affiliations:** 1 Department of Clinical Pharmacology, King Abdulaziz University Faculty of Medicine, Jeddah, SAU; 2 Preventive Medicine, College of Medicine, Jeddah University, Jeddah, SAU; 3 Department of Hematology, King Abdulaziz University Faculty of Medicine, Jeddah, SAU; 4 Section of Infectious Diseases, Department of Medicine, King Faisal Specialist Hospital and Research Centre, Jeddah, SAU; 5 Department of Medicine, King Abdulaziz University Faculty of Medicine, Jeddah, SAU; 6 Department of Computer Science, Faculty of Computing and Information Technology, King Abdulaziz University, Jeddah, SAU; 7 Department of Information Systems, Faculty of Computing and Information Technology, King Abdulaziz University, Jeddah, SAU; 8 Intensive Care Unit, King Fahad General Hospital, Jeddah, SAU; 9 Hematology Section, Medical Department, King Fahad General Hospital, Jeddah, SAU; 10 Department of Medicine, Faculty of Medicine, Duke University, Durham, USA

This article has been retracted by the Editor-in-Chief due to major flaws which undermine the credibility of the article. The article reports biologically impossible hematology values used in modeling, including hemoglobin up to 116 g/dL and neutrophil percentages exceeding 100. Multiple models also report mathematically incompatible performance metrics, such as near-perfect sensitivity with zero or very low F1 scores, or AUC values below 0.5 alongside high accuracy and sensitivity. The ROC results themselves are internally inconsistent, showing near no-skill performance while strong classification metrics are simultaneously reported. Finally, the strikingly uniform, near-identical AUCs across fundamentally different model families further undermine the credibility of the reported model performance. The authors disagree with the retraction.

